# Technical Specification for Hemocompatibility Assessment of Human Mesenchymal Stem Cells

**DOI:** 10.1111/cpr.70183

**Published:** 2026-03-12

**Authors:** Jialing Liu, Weiyang Liang, Wenjie Xia, Ping Wang, Jie Hao, Tao Na, Sunxing Yan, Yu Zhang, Ka Li, Qiyuan Li, Guangjin Pan, Jun Wei, Qubo Chen, Jiani Cao, Peijun Zhai, Boqiang Fu, Hengjun Gao, Yong Zhang, Lei Wang, Meimei Guo, Shijun Hu, Lijun Zhu, Yang Zhao, Weiqi Zhang, Zhihong Wu, Yifang Ping, Hongling Zhao, Shuaishuai Niu, Tongbiao Zhao, Aijin Ma, Andy Peng Xiang, Xiaoyong Chen

**Affiliations:** ^1^ Center for Stem Cell Biology and Tissue Engineering, Key Laboratory for Stem Cells and Tissue Engineering, Ministry of Education, Sun Yat‐Sen University Guangzhou China; ^2^ NMPA Key Laboratory for Quality Control of Blood Products, Guangdong Institute for Drug Control Guangzhou China; ^3^ Institute of Blood Transfusion, Guangzhou Blood Centre Guangzhou China; ^4^ Shenzhen Institute for Drug Control, Shenzhen Key Laboratory of Drug Quality Standard Research Shenzhen China; ^5^ National Stem Cell Resource Center, Institute of Zoology, Chinese Academy of Sciences Beijing China; ^6^ Standard Committee, Chinese Society for Cell Biology Shanghai China; ^7^ National Institutes for Food and Drug Control Beijing China; ^8^ Guangzhou Cellgenes Biotechnology Co., Ltd. Guangzhou China; ^9^ Zephyrm Biotechnologies Co., Ltd Beijing China; ^10^ Zhongshan Hospital, Fudan University Shanghai China; ^11^ Shenzhen Medical Academy of Research and Translation Shenzhen China; ^12^ Guangzhou Institutes of Biomedicine and Health, Chinese Academy of Sciences Guangzhou China; ^13^ General Hospital of Ningxia Medical University Ningxia China; ^14^ State Key Laboratory of Dampness Syndrome of Chinese Medicine, the Second Affiliated Hospital of Guangzhou University of Chinese Medicine Guangzhou China; ^15^ China National Accreditation Service for Conformity Assessment (CNAS) Beijing China; ^16^ National Institute of Metrology Beijing China; ^17^ National Engineering Center for Biochip at Shanghai Shanghai China; ^18^ Shenzhen HHLIFE Co., Ltd. Shenzhen China; ^19^ Shenzhen Qianhai Shekou Free Trade Zone Hospital Shenzhen China; ^20^ Department of Cardiovascular Surgery of the First Affiliated Hospital & Institute for Cardiovascular Science, Medical College, Soochow University Suzhou China; ^21^ Institute of Scientific and Technical Information of China Beijing China; ^22^ Peking University China; ^23^ China National Center for Bioinformation and Beijing Institute of Genomics, Chinese Academy of Sciences Beijing China; ^24^ Peking Union Medical College Hospital, Chinese Academy of Medical Sciences Beijing China; ^25^ Army Military Medical University Chongqing China; ^26^ Beijing Institute for Stem Cell and Regenerative Medicine Beijing China; ^27^ Beijing Technology and Business University Beijing China

## Abstract

‘Technical specification for haemocompatibility assessment of human mesenchymal stem cells’ is the first set of guidelines on haemocompatibility assessment of human mesenchymal stem cells (MSCs) in China, jointly drafted and agreed upon by experts from the Chinese Society for Stem Cell Research. This standard outlines the methods for assessing the hemocompatibility of human mesenchymal stem cells and specifies the requirements for the selection of evaluation indicators, calculation of indicator values, and determination of hemocompatibility levels. It is applicable for evaluating the hemocompatibility of MSCs prior to their contact with blood. This guideline was originally released by the Chinese Society for Cell Biology on October 28, 2024. We anticipate that the publication of this specification will promote the institutional adoption, acceptance, and execution of proper testing protocols, thereby accelerating the international standardization of MSCs for clinical development and therapeutic applications.

## Graphical abstract

A comprehensive evaluation of MSC haemocompatibility is a critical prerequisite for ensuring the safety of systemic delivery. A haemocompatibility level of ‘poor’ indicates a high probability of adverse blood reactions when MSCs contact with blood.

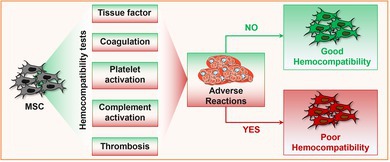

## 1 Scope

This document outlines the methods for assessing the haemocompatibility of human mesenchymal stem cells (MSCs) and specifies the requirements for selecting evaluation indicators, calculating indicator values, and determining haemocompatibility levels.

This document is applicable for evaluating the haemocompatibility of MSCs prior to their contact with blood.

## 2 Normative References

The following documents contain provisions that, through normative references in this document, constitute essential clauses. For documents marked with a date, only the version corresponding to that date applies; for documents without a date, the latest version (including all amendments) applies.

GB/T 6682. *Water for analytical laboratory use—Specification and test methods*.

T/CSCB 0001–2020. *General Requirements for Stem Cells*.

T/CSCB 0003–2021. *Human Mesenchymal Stem Cells*.

*Pharmacopoeia of the People's Republic of China*.

*National Guide to Clinical Laboratory Procedures*.

## 3 Terms and Definitions

The following terms and definitions apply to this document.

### 3.1 Mesenchymal Stem Cells

A type of stem cell exhibits a fibroblast‐like morphology (spindle and fusiform) after adherent culture and possesses the ability to self‐renew and differentiate into the osteogenic, adipogenic, and chondrogenic lineages in vitro.

[Source: T/CSCB 0003–2021] [3].

### 3.2 Tissue Factor (TF)

A membrane glycoprotein receptor that is the primary initiator of the coagulation response, capable of activating the extrinsic coagulation pathway by activating factor VII, which in turn activates factor X, leading to thrombin activation and the formation of fibrin.

### 3.3 Coagulation

The phenomenon of blood clotting resulting from the cascade activation of coagulation factors.

### 3.4 Haemocompatibility

The property of MSCs in contact with blood that does not induce any significant adverse effects, such as coagulation, platelet activation, complement activation, and/or other blood‐related adverse events.

[Note 1 to entry: According to ISO 10993, haemocompatibility is defined as the acceptable response of blood to foreign substances or materials].

[Note 2 to entry: Based on the definition in GB/T 16886.4‐2022, the term ‘haemocompatibility’ specifically refers to the interaction between ‘blood’ and ‘mesenchymal stem cells’] [4].

### 3.5 Blood/Mesenchymal Stem Cells Interaction

The interaction between blood or its components and mesenchymal stem cells.

### 3.6 Platelet

Anucleate cell fragments present in blood that contribute to thrombosis formation through adhesion, release of factors, and/or aggregation to form thrombi.

### 3.7 Thrombus

A coagulated mixture composed of red blood cells, aggregated platelets, fibrin, and other cellular components.

### 3.8 Thrombosis

The formation of a thrombus is caused by the activation of the coagulation system and platelets in flowing whole blood, occurring under in vivo or in vitro simulated conditions.

## 4 Abbreviations

The following abbreviations apply to this document:

CD: cluster of differentiation.

β‐TG: β‐thromboglobulin.

PF4: platelet factor 4.

SC5b‐9: soluble terminal complement complex.

TAT: thrombin‐antithrombin complex.

TF: tissue factor.

TxB2: thromboxane B2.

## 5 General Principles

### 5.1 Scientificity

A stable evaluation system should be established to accurately reflect the expression levels of tissue factor in MSCs, as well as changes in coagulation, platelet activation, complement activation, and in vivo thrombosis formation.

### 5.2 Well‐Targeted

Haemocompatibility assessment is selectively performed based on the application needs of MSCs. Haemocompatibility assessments should be performed for MSCs involved in contact with blood.

### 5.3 Normative

The MSCs used for evaluation must be collected and produced in accordance with T/CSCB 0001‐2020 and T/CSCB 0003‐2021, and key quality attributes shall comply with the relevant requirements of T/CSCB 0003‐2021.

### 5.4 Comprehensiveness

Both in vivo and in vitro assessments shall be conducted.

### 5.5 Authenticity

Assessment data shall be truthful, and results shall be reliable.

## 6 Core Technical Elements

### 6.1 Evaluation Indicators

As shown in Table 1, the evaluation indicators consist of eight assessment factors within five categories, including protein expression, coagulation tests, platelet activation tests, complement activation tests, and thrombosis tests.

**TABLE 1:** Evaluation indicators and their selection.

Evaluation category		Evaluation indicators		
Name	Code	Indicator name	Code	Recommendation
Protein level	A	Tissue factor	A1	Required
Coagulation test	B	TAT	B1	Required
Platelet activation test	C	β‐TG	C1	Required
		PF4	C2	Required
		TxB2	C3	Optional
Complement activation Test	D	SC5b‐9	D1	Required
		C3a	D2	Optional
Thrombosis test	E	In vivo thrombosis	E1	Required

### 6.2 Evaluation Methods

#### 6.2.1 Scope of Evaluation

The MSCs to be evaluated can be isolated from multiple tissues (such as bone marrow, umbilical cord, placenta, adipose tissue, umbilical cord blood, among others), and can also be obtained through differentiation or transdifferentiation.

#### 6.2.2 Selection of Evaluation Indicators

The selection of evaluation indicators is detailed in Table 1.

#### 6.2.3 Calculation Methods and Data Sources

Calculation methods and data sources for evaluating the haemocompatibility of MSCs are determined according to the requirements in Table 2, and relevant detection data should be recorded, stored, and maintained.

**TABLE 2:** Calculation methods and data sources for evaluation factors.

Evaluation factor and code	Calculation method	Data source
Tissue factor (A1)	Percentage of TF positive cells out of total cells	According to Annex A
TAT (B1)	Ratio of TAT in test sample to TAT in control sample (percentage)	According to Annex B
β‐TG (C1)	Ratio of β‐TG in test sample to β‐TG in control sample (percentage)	According to Annex C
PF4 (C2)	Ratio of PF4 in test sample to PF4 in control sample (percentage)	According to Annex C
TxB2 (C3)	Ratio of TxB2 in test sample to TxB2 in control sample (percentage)	According to Annex C
SC5b‐9 (D1)	Ratio of SC5b‐9 in test sample to SC5b‐9 in control sample (percentage)	According to Annex D
C3a (D2)	Ratio of C3a in test sample to C3a in control sample (percentage)	According to Annex D
In vivo thrombosis (E1)	Count of thrombi in tested tissue	According to Annex E

### 6.3 Determining Haemocompatibility Levels

#### 6.3.1 Classification and Determination of Compatibility Levels

Compatibility levels are classified as poor or good. According to the calculation of evaluation factors, the compatibility level of each evaluation factor can be determined, as shown in Table 3.

**TABLE 3:** Determination of compatibility levels for evaluation factors.

Evaluation factor and code	Compatibility level	
	Poor	Good
TF (A1)	TF expression rate > 10%	TF expression rate ≤ 10%
TAT (B1)	Ratio ≤ 80% or ≥ 120%	80% < Ratio < 120%
β‐TG (C1)	Ratio ≤ 80% or ≥ 120%	80% < Ratio < 120%
PF4 (C2)	Ratio ≤ 80% or ≥ 120%	80% < Ratio < 120%
TxB2 (C3)	Ratio ≤ 80% or ≥ 120%	80% < Ratio < 120%
SC5b‐9 (D1)	Ratio ≤ 80% or ≥ 120%	80% < Ratio < 120%
C3a (D2)	Ratio ≤ 80% or ≥ 120%	80% < Ratio < 120%
In Vivo Thrombosis (E1)	Thrombus count ≥ 1	Thrombus count = 0

#### 6.3.2 Determining Haemocompatibility Level of Human Mesenchymal Stem Cells

The lowest compatibility level among all evaluation factors is determined as the haemocompatibility level of human mesenchymal stem cells.

Note 1 to entry: A haemocompatibility level of ‘poor’ indicates a high probability of adverse blood reactions when MSCs contact with blood, necessitating careful selection or relevant countermeasures based on the specific circumstances.

## 7 Evaluation Report

The evaluation report should include at least the following content:

- Basic information about the assessed mesenchymal stem cells (including but not limited to source, viability, etc.);
- Basic information about the control samples (cells) used for evaluation:
- Selection of evaluation indicators and evaluation methods:
- Determination of compatibility levels for evaluation factors:
- Evaluation results and related explanations/recommendations.

## Author Contributions

Chen X., Xiang A.P., Zhao T. and Ma A. contributed to conception and design. Chen X., Xiang A.P., Liu J., Liang W. and Xia W. drafted and revised the manuscript. Na T., Yan S., Zhang Yu, Wang C., Li K., Li Q., Pan G., Wei J., Chen Q., Cao J., Zhai P., Fu B., Gao H., Zhang Y., Wang L., Guo M., Hu S., Zhu L., Zhao Y., Zhang W., Wu Z., Ping Y., Zhao H. and Niu S. critically read and revised the manuscript.

## Funding

This work was supported by National Key Research and Development Program of China Stem Cell and Translational Research, 2022YFA1104100, 2022YFA1105000; National Natural Science Foundation of China, 82280230, 32130046, 82570270; Guangdong Basic and Applied Basic Research Foundation, 2023B1515020119; Key Scientific and Technological Program of Guangzhou City, 2023B01J1002; Shenzhen Science and Technology Program, KJZD20230923114504008; Sanming Project of Medicine in Shenzhen Nanshan, SZSM202103012; Special Funds for the Cultivation of Guangdong College Students' Scientific and Technological Innovation, pdjh2025ak003.

## Supporting information


**Data S1:** Annex A (normative) Tissue factor protein expression (flow cytometry).
Annex B (normative) Coagulation test (Thrombin–Antithrombin complex (TAT) assay).
Annex C (normative) Platelet activation assay (detection of β‐TG, PF4, and TxB2).
Annex D (normative) Complement activation assay (detection of SC5b‐9 and C3a).
Annex E (normative) In vivo thrombosis formation test (rat model).

## Data Availability

The data that support the findings of this study are available from the corresponding author upon reasonable request.
