# Supplementary material for: Technical Specification for Hemocompatibility Assessment of Human Mesenchymal Stem Cells
Source: Cell Prolif. 2026 Mar 12;59(4):e70183. doi: 10.1111/cpr.70183 (PMC13051868; doi:10.1111/cpr.70183)
Supplement: Supplementary file 1 — Data S1: Annex A (normative) Tissue factor protein expression (flow cytometry). Annex B (normative) Coagulation test (Thrombin–Antithrombin complex (TAT) assay). Annex C (normative) Platelet activation assay (detection of β‐TG, PF4, and TxB2). Annex D (normative) Complement activation assay (detection of SC5b‐9 and C3a). Annex E (normative) In vivo thrombosis formation test (rat model). [file CPR-59-e70183-s001.docx]

**Annex A** **(Normative)** **Tissue Factor Protein Expression (Flow cytometry)**

**A.1 General Principles**

Tissue factor (CD142) is a membrane protein that is a component of the factor VIIa-TF complex, playing a crucial role in physiological hemostasis and thrombosis formation. Upon vascular injury, tissue factor is exposed to blood and binds to plasma factor VIIa, resulting in a complex that initiates a cascade of enzymatic reactions leading to coagulation and thrombus formation. Therefore, detecting CD142 molecules on the surface of human mesenchymal stem cells (MSCs) using flow cytometry is significant for identifying positive cell populations and assessing tissue factor expression.

**A.2 Instruments and Equipment**
A.2.1 Flow cytometer.
A.2.2 Centrifuge (horizontal rotor).
A.2.3 Electronic balance.

**A.3 Reagents**

Unless otherwise stated, all the reagents used shall be analytical grade. The water used in the experiment shall be Grade 1 water as stipulated in GB/T 6682.
A.3.1 Phosphate-buffered saline (PBS), pH 7.4.
A.3.2 Bovine serum albumin (BSA): purity ≥ 98%.
A.3.3 Sodium azide (NaN₃).
A.3.4 Anti-human CD142 antibody and isotype control antibody.
A.3.5 Prepare the required solutions for flow cytometry (e.g., wash buffer, antibody dilution buffer) using the electronic balance (A.2.3) according to the specifications.

**A.4 Sample Storage**

The wash solution and labeled samples shall be stored at 2 °C to 8 °C. Antibodies shall be stored according to the manufacturer’s instructions.

**A.5 Testing protocol**
A.5.1 Sample Preparation
Collect fresh, adherent cultured human mesenchymal stem cells and human umbilical vein endothelial cells by centrifuging single cell suspensions with Centrifuge (horizontal rotor) (A.2.2) at 300×g for 4 minutes, and discard the supernatant. Wash the cell samples with an appropriate volume of wash solution, then centrifuge again at 300×g for 4 minutes and discard the supernatant. Resuspend the cells in phosphate-buffered saline (A.3.1) to a concentration of 1×10⁶ cells/mL.

A.5.2 Experimental Grouping

a) Negative Control: 100 µL human umbilical vein endothelial cells + anti-CD142 antibody.

b) Test Samples: 100 µL human mesenchymal stem cells + anti-CD142 antibody.

c) Isotype Control 1: 100 µL human umbilical vein endothelial cells + isotype control antibody.

d) Isotype Control 2: 100 µL human mesenchymal stem cells + isotype control antibody.

A.5.3 Antibody Incubation

For each experimental group, take an appropriate volume of the cell suspension and add the recommended dilution of flow cytometry antibodies according to the manufacturer’s instructions. For isotype control groups, add an equal volume of isotype control antibodies. Incubate the samples under conditions recommended by manufacturer. After incubation, wash the cells twice with wash buffer, centrifuge at 300×g for 4 minutes, and discard the supernatant.

A.5.4 Cell Filtration and detection

Resuspend the cells in wash buffer, then transfer them to flow cytometry tubes through a 40 µm filter. Load the samples into the flow cytometer and perform detection according to the manufacturer’s instruction.

A.5.5 Gating

First, gate on the target cell population based on particle size and granularity, excluding dead cells and other debris. Then, the gating of positive staining cells shall be determined by the fluorescence intensity using isotype controls as a reference. Both positive and negative experimental controls shall be set up for gating and the following analysis.

**A.6 Result Analysis**

Analyze the results using software according to manufacturer’s instructions.

**A.7 Result Evaluation**
A.7.1 The result for human umbilical vein endothelial cells should be negative, with TF expression rate ≤ 2%.
A.7.2 Evaluate tissue factor expression in human mesenchymal stem cells based on the TF expression rate:

a) Negative Expression: TF expression rate ≤ 2%

b) Low Expression: 2% < TF expression rate ≤ 10%

c) Moderate Expression: 10% < TF expression rate ≤ 25%

d) High Expression: TF expression rate > 25%

**Annex B** **(Normative)** **Coagulation Test (Thrombin-Antithrombin Complex (TAT) Assay)**

**B.1 General Principles**
The thrombin-antithrombin complex (TAT) is a product of the interactions between coagulation and anticoagulation, maintaining physiological balance in the human body. It serves as one of the biomarkers for thrombin generation. Following the formation of thrombin in vivo, a portion thrombin rapidly binds to antithrombin (AT) to form the TAT complex. This indicator reflects thrombin generation and can sensitively assess the activation level of the coagulation system, thereby directly indicating the initiation of the coagulation response.

**B.2 Instruments and Equipment**
B.2.1 Enzyme-linked immunosorbent assay (ELISA) reader.
B.2.2 Centrifuge (horizontal rotor).
B.2.3 Water bath.

**B.3 Reagents**
Unless otherwise stated, all the reagents used shall be analytical grade. The water used in the experiment shall be Grade 1 water as stipulated in GB/T 6682.
B.3.1 Phosphate-buffered saline (PBS), pH 7.4.
B.3.2 TAT detection kit (ELISA).

**B.4 Sample Storage**
Phosphate-buffered saline should be stored at 2 °C to 8 °C. The reagent kit should be stored according to the manufacturer’s instructions.

**B.5 Testing protocol**
B.5.1 Sample Preparation
B.5.1.1 Collect fresh, non-anticoagulated whole blood from healthy volunteers (informed consent obtained and no medication received for at least 10 days). Centrifuge using a horizontal centrifuge (B.2.2) at 2500×g for 10 minutes at 20-22 °C. Collect the plasma and store at room temperature (not exceeding 8 hours).
B.5.1.2 Collect fresh, adherent cultured human mesenchymal stem cells and human umbilical vein endothelial cells. Centrifuge using a horizontal centrifuge (B.2.2) at 300×g for 4 minutes, then discard the supernatant. Wash once with wash buffer, then centrifuge again at 300×g for 4 minutes and discard the supernatant. Resuspend the cells in phosphate-buffered saline (B.3.1) to a concentration of 1×10⁷ cells/mL.

B.5.2 Experimental Grouping

a) Blank Control: In a 1.5 mL centrifuge tube, mix 50 μL of phosphate-buffered saline with 500 μL of plasma, preparing three parallel samples.

b) Negative Control: In a 1.5 mL centrifuge tube, mix 50 μL of human umbilical vein endothelial cell suspension with 500 μL of plasma, preparing three parallel samples.

c) Test Samples: In a 1.5 mL centrifuge tube, mix 50 μL of mesenchymal stem cell suspension with 500 μL of test plasma, preparing three parallel samples.

B.5.3 Sample Incubation
Take the appropriate volumes of cell suspension and plasma according to the experimental groups, mix thoroughly, and incubate in a water bath (B.2.3) at 37 °C for 30 minutes. After incubation, retain samples for detection according to the TAT kit instructions.

B.5.4 TAT Detection
Conduct the experiment following the manufacturer's instructions provided with the kit. Use the ELISA reader (B.2.1) according to the recommended method in the kit instructions.

**B.6 Data Analysis**
Construct a standard curve as required by the kit, calculating the r² value and the equation of the fitted curve. Calculate the TAT content for each sample, determining the average values A (blank control), B (negative control), and C (test samples).

Calculate the percentage of the negative control relative to the blank control using the formula:
D1 = (B/A) × 100% ……………………………… (B.1)
In this equation:
D1 = percentage of negative control relative to blank control;
A = average value of blank control;
B = average value of negative control.

Calculate the percentage of the test samples relative to the blank control using the formula:
D2 = (C/A) × 100% ……………………………… (B.2)
In this equation:
D2 = percentage of test samples relative to blank control;
A = average value of blank control;
C = average value of test samples.

**B.7 Result Evaluation**
B.7.1 D1 should range between 80% and 100%; otherwise, the experimental results are unacceptable.
B.7.2 For D2, the negative range is defined as 80% < ratio < 120%; positive results are indicated by a ratio ≤ 80% or ≥ 120%.

**Annex C** **(Normative)** **Platelet Activation Assay (Detection of β-TG, PF4, and TxB2)**

**C.1 General Principles**
β-thromboglobulin (β-TG) and platelet factor 4 (PF4) are specific proteins contained in platelet alpha granules. When platelets are activated by certain bioactive substances such as thrombin, epinephrine, ADP, and thromboxane A2 (TxA2), a significant amount of β-TG and PF4 is released from the platelets into the surrounding plasma. Measuring the levels of β-TG and PF4 in plasma can be utilized to study platelet release function and reflect the activation status of platelets. Additionally, TxB2 is a metabolic product of thromboxane A2 (TxA2) and prostacyclin (PGI2). Under physiological conditions, TxA2 and PGI2 maintain a relative balance, which is crucial for ensuring smooth blood flow. An increase in TxA2 disrupts this balance, promoting platelet aggregation within blood vessels and ultimately leading to thrombosis. Due to the instability of TxA2, the elevated levels of its metabolite TxB2 are significant for assessing platelet formation.

**C.2 Instruments and Equipment**
C.2.1 Enzyme-linked immunosorbent assay (ELISA) reader.
C.2.2 Centrifuge (horizontal rotor).
C.2.3 Water bath.

**C.3 Reagents**
Unless otherwise stated, all the reagents used shall be analytical grade. The water used in the experiment shall be Grade 1 water as stipulated in GB/T 6682.
C.3.1 Phosphate-buffered saline (PBS), pH 7.4.
C.3.2 β-TG detection kit (ELISA).
C.3.3 PF4 detection kit (ELISA).
C.3.4 TxB2 detection kit (ELISA).

**C.4 Sample Storage**
Phosphate-buffered saline should be stored at 2 °C to 8 °C. The reagent kits should be stored according to the manufacturer’s instructions.

**C.5 Testing protocol**
C.5.1 Sample Preparation
C.5.1.1 Collect fresh, anticoagulated whole blood from healthy volunteers (informed consent obtained, and no medications received for at least 10 days). Invert the collection tube to mix thoroughly and store at room temperature for later use.
C.5.1.2 Collect fresh, adherent cultured human mesenchymal stem cells and human umbilical vein endothelial cells. Centrifuge using a horizontal centrifuge (C.2.2) at 300×g for 4 minutes, then discard the supernatant. Wash the cells once with wash buffer, then centrifuge again at 300×g for 4 minutes and discard the supernatant. Resuspend the cells in phosphate-buffered saline (C.3.1) to a concentration of 1×10⁷ cells/mL.

C.5.2 Experimental Grouping

a) Blank Control: Phosphate-buffered saline + anticoagulated whole blood, with three parallel samples.

b) Negative Control: Human umbilical vein endothelial cell suspension + anticoagulated whole blood, with three parallel samples.

c) Test Samples: Human mesenchymal stem cell suspension + anticoagulated whole blood, with three parallel samples.

**C.5.3 Sample Incubation**
For each experimental group, take an appropriate volume of the cell suspension and add anticoagulated whole blood in the proportions recommended by the ELISA kit manufacturer. Incubate all tubes in a water bath at 37 °C while shaking for 1 hour at a speed of 60 rpm. After incubation, add EDTA to each tube to a final concentration of 5 mmol/L to terminate the reaction. Gently mix each tube, then transfer the entire blood contents to a corresponding clean tube. Centrifuge at 1500×g for 10 minutes and collect the upper platelet-poor plasma (PPP) for detection.

**C.5.4 Detection of β-TG, PF4, and TxB2**
Conduct the experiments according to the instructions provided by the kit manufacturer. Use the ELISA reader (C.2.1) following the recommended methods in the kit documentation for detection.

**C.6 Data Analysis**
Construct a standard curve as required by the kit, calculating the r² value and the equation of the fitted curve. Calculate the β-TG, PF4, and TxB2 concentrations for each sample, determining the average values: A (blank control), C (negative control), and C (test samples).

Calculate the percentage of the negative control relative to the blank control using the formula:
D1 = (C/A) × 100% ……………………………………… (C.1)
In this equation:
D1 = percentage of negative control relative to blank control;
A = average value of blank control;
C = average value of negative control.

Calculate the percentage of the test samples relative to the blank control using the formula:
D2 = (C/A) × 100% ……………………………………… (C.2)
In this equation:
D2 = percentage of test samples relative to blank control;
A = average value of blank control;
C = average value of test samples.

**C.7 Result Evaluation**
C.7.1 The range of D1 for β-TG, PF4, or TxB2 should be between 80% and 100%; otherwise, the experimental results are unacceptable.
C.7.2 The negative range for D2 of β-TG, PF4, or TxB2 is defined as 80% < ratio < 120%; positive results are indicated by a ratio ≤ 80% or ≥ 120%.

**Annex D** **(Normative)** **Complement Activation Assay (Detection of SC5b-9 and C3a)**

**D.1 General Principles**
The activation of the complement system is a highly ordered cascade reaction, regulated by a series of complex factors. SC5b-9 is a hydrophilic, non-cell-activating macromolecular fragment that forms when the common terminal product C5b-9 of the complement system interacts with the S protein in serum. C3a is a small molecule generated upon the activation of complement component C3. By measuring the levels of SC5b-9 or C3a, the activation status of the complement system in serum can be assessed.

**D.2 Instruments and Equipment**
D.2.1 Enzyme-linked immunosorbent assay (ELISA) reader.
D.2.2 Centrifuge (horizontal rotor).
D.2.3 Water bath.

**D.3 Reagents**
Unless otherwise stated, all the reagents used shall be analytical grade. The water used in the experiment shall be Grade 1 water as stipulated in GB/T 6682.
D.3.1 Phosphate-buffered saline (PBS), pH 7.4.
D.3.2 SC5b-9 detection kit (ELISA).

**D.4 Sample Storage**
Phosphate-buffered saline should be stored at 2 °C to 8 °C. The reagent kits should be stored according to the manufacturer’s instructions.

**D.5 Testing Protocol**
D.5.1 Sample Preparation
D.5.1.1 Collect fresh, non-anticoagulated whole blood from healthy volunteers (informed consent obtained and no medications received for at least 10 days). Allow the blood to stand at 4 °C for 30 minutes, then centrifuge at 1500×g for 10 minutes at 4 °C. Collect the upper serum layer and store it on ice for later use.
D.5.1.2 Collect fresh, adherent cultured human mesenchymal stem cells and human umbilical vein endothelial cells. Centrifuge using a horizontal centrifuge (D.2.2) at 300×g for 4 minutes, then discard the supernatant. Wash the cells once with wash buffer, then centrifuge again at 300×g for 4 minutes and discard the supernatant. Resuspend the cells in phosphate-buffered saline (D.3.1) to a concentration of 1×10⁷ cells/mL.

D.5.2 Experimental Grouping

a) Blank Control: Phosphate-buffered saline + serum, with three parallel samples.

b) Negative Control: Human umbilical vein endothelial cell suspension + serum, with three parallel samples.

c) Test Samples: Human mesenchymal stem cell suspension + serum, with three parallel samples.

**D.5.3 Sample Incubation**
For each experimental group, take an appropriate volume of the cell suspension and add serum in the proportions recommended by the ELISA kit manufacturer. Incubate all tubes in a water bath at 37 °C while shaking for 1 hour at a speed of 60 rpm. After incubation, gently mix all tubes, transfer the contents to corresponding labeled clean tubes, and store them on ice.

**D.5.4 Detection of SC5b-9 or C3a**
Conduct the experiments according to the instructions provided by the kit manufacturer. Use the ELISA reader (D.2.1) following the recommended methods in the kit documentation for detection.

**D.6 Data Analysis**
Construct a standard curve as required by the kit, calculating the r² value and the equation of the fitted curve. Calculate the concentrations of SC5b-9 or C3a for each sample, determining the average values: A (blank control), C (negative control), and C (test samples).

Calculate the percentage of the negative control relative to the blank control using the formula:
D1 = (C/A) × 100% ……………………………………… (D.1)
In this equation:
D1 = percentage of negative control relative to blank control;
A = average value of blank control;
C = average value of negative control.

Calculate the percentage of the test samples relative to the blank control using the formula:
D2 = (C/A) × 100% ……………………………………… (D.2)
In this equation:
D2 = percentage of test samples relative to blank control;
A = average value of blank control;
C = average value of test samples.

**D.7 Result Evaluation**
D.7.1 The range of D1 for SC5b-9 or C3a should be between 80% and 100%; otherwise, the experimental results are unacceptable.
D.7.2 The negative range for D2 of SC5b-9 or C3a is defined as 80% < ratio < 120%; positive results are indicated by a ratio ≤ 80% or ≥ 120%.

**Annex E** **(Normative)** **In Vivo Thrombosis Formation Test (Rat Model)**

**E.1 General Principles**
In clinically relevant animal models, in vivo thrombosis detection plays an important role in assessing blood safety. Reports have indicated a risk of thrombosis following the intravenous infusion of mesenchymal stem cells (MSCs). Due to hemodynamic factors, thrombi tend to accumulate in terminal organs such as the lungs, liver, spleen, and kidneys. Therefore, observing thrombosis formation in these organs is beneficial for evaluating whether tissue factor from MSCs mediates thrombosis.

**E.2 Instruments and Equipment**
E.2.1 Microscope.
E.2.2 Centrifuge (horizontal rotor).
E.2.3 Microtome.
E.2.4 Electronic balance.

**E.3 Reagents**
Unless otherwise stated, all the reagents used shall be analytical grade. The water used in the experiment shall be Grade 1 water as stipulated in GB/T 6682.
E.3.1 Phosphate-buffered saline (PBS), pH 7.4.
E.3.2 1% Pentobarbital Sodium.
E.3.3 Embedding medium.
E.3.4 Hematoxylin and Eosin (H&E).

**E.4 Sample Storage**
Phosphate-buffered saline should be stored at 2 °C to 8 °C. Reagents should be stored according to the manufacturer’s instructions.

**E.5 Testing Protocol**
E.5.1 Sample Preparation
Collect fresh, adherent cultured human mesenchymal stem cells. Centrifuge using a horizontal centrifuge (E.2.2) at 300×g for 4 minutes, then discard the supernatant. Wash the cells once with wash buffer, then centrifuge again at 300×g for 4 minutes and discard the supernatant. Resuspend the cells in phosphate-buffered saline (E.3.1) to a concentration of 1×10⁷ cells/mL.

E.5.2 Experimental Grouping
SPF-grade, healthy, adult rats (e.g., F344 rats) are grouped as follows:

a) Blank Control: Infused with phosphate-buffered saline, 3-5 rats.

b) Test Samples: Infused with human mesenchymal stem cell suspension, 3-5 rats.

E.5.3 Procedure
Weigh the SPF-grade, normally fed rats and randomly group them. Using a sterile 1 mL syringe, inject each rat via the tail vein with 100 μL of either 6×10⁶/kg human mesenchymal stem cell suspension or 100 μL of phosphate-buffered saline. Start timing immediately after injection for 1 hour. Following this, anesthetize the rats with 1% pentobarbital sodium, perform dissection, and conduct cardiac perfusion. Isolate and remove the lungs, liver, spleen, and kidneys of the rats, and fix the tissues in 4% paraformaldehyde (PFA) for 24 hours.

E.5.4 Staining and Observation
Embed the tissues and prepare paraffin sections. Stain with hematoxylin and eosin according to the reagent instructions. Randomly select 5 fields of view for each tissue from each rat and observe for thrombus formation in the blood vessels of the lungs, liver, spleen, and kidneys under a microscope, documenting the findings with photographs.

**E.6 Result Evaluation**
Assess thrombosis formation by counting thrombi:

a) Negative: No thrombi detected in any of the examined tissues.

b) Positive: Thrombi detected in any of the examined organs.
